# Curdione Plays an Important Role in the Inhibitory Effect of *Curcuma aromatica* on CYP3A4 in Caco-2 Cells

**DOI:** 10.1093/ecam/nep229

**Published:** 2011-03-13

**Authors:** Xiao-Long Hou, Emi Hayashi-Nakamura, Tomoka Takatani-Nakase, Ken Tanaka, Kyoko Takahashi, Katsuko Komatsu, Koichi Takahashi

**Affiliations:** ^1^Department of Pharmaceutics, School of Pharmaceutical Sciences, Mukogawa Women's University, 9-11-68 koushien, Nishinomiya City, Hyogo, Japan; ^2^Division of Pharmacognosy, Department of Medicinal Resources, Institute of Natural Medicine, University of Toyama, 2630 Sugitani, Toyama City, Toyama, Japan; ^3^Department of Medicinal Resources, Graduate School of Pharmaceutical Science, Osaka University, 1-6 Yamadaoka, Suita City, Osaka, Japan

## Abstract

*Curcuma aromatica* is a plant belonging to genus *Curcuma* of family *Zingiberaceae* and is widely used as supplements in Japan. Rhizomes of *C. aromatica* have curcumin as a major yellow pigment and curdione as a main ingredient of essential oils. In this study, we investigated the affect of *C. aromatica* on CYP3A4 using 1**α**,25-(OH)_2_-D_3_-treated Caco-2 clone cells. Caco-2 cells were treated with methanol extract (0.1 mg ml^−1^), its hexane soluble fraction (0.1 mg ml^−1^), curcumin (4 **μ**M) and curdione (20 **μ**M) for 72 hours. Nifedipine was used as a substrate of CYP3A4. Methanol extract, hexane fraction and curdione inhibited the formation of oxidized nifedipine by 50–70%, and curcumin showed no effect. The IC50s of methanol extract, hexane fraction and curdione to oxidized nifedipine formation were 21, 14 and 3.9 **μ**g ml^−1^ (16.9 **μ**M), respectively. The content of curdione in methanol extract was 11.4%. Moreover, all of methanol extract, hexane fraction and curdione decreased CYP3A4 protein expression but had no affect on CYP3A4 mRNA expression. Our results showed that these drugs further decreased the CYP3A4 protein expression level after the protein synthesis was inhibited by cychroheximide. These findings suggest that curdione plays an important role in the CYP3A4 inhibitory activity of *C. aromatica* and curdione might inhibit the activity by accelerating the degradation of CYP3A4.

## 1. Introduction

**Figure 1:**
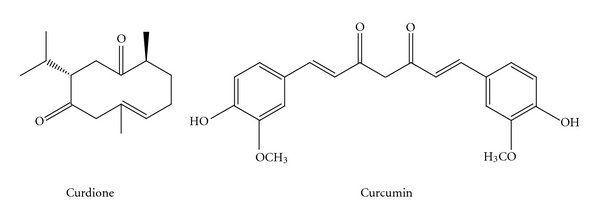
Chemical structure of curcumin and curdione.

*Curcuma aromatica* (also called Haruukon in Japanese) is a plant belonging to the genus *Curcuma* of family *Zingiberaceae.* In Japan, *C. aromatica* is called *Spring Curcuma* because it is in bloom in April/May and *C. longa* is called *Autumn Curcuma* because it is in bloom in July/August. Traditionally, *C. longa* is recommended to improve hepatica function and *C. aromatica* is administrated to people with gastrointestinal dysfunction. In present, both *C. aromatica* and *C. longa* are used as supplements because of their various pharmacological activities including promotion of blood circulation, anti-cancer and anti-inflammation [1, 2]. Curcumin [1,7-*bis*(4-hydroxy-3-methoxyphenyl)-1,6-heptadiene-3,5-dione; Figure 1) is a yellow pigment from *C. aromatica* and *C. longa*. It has been reported that curcumin possesses wide range of pharmacological activities and is widely used, especially as a cancer chemopreventive agent [3, 4]. Salvioli et al. [5] discussed the multitudinous biological mechanisms and possible clinical effects of curcumin and put forward a xenohormetic mechanism. Normally, the content of curcumin in *C. longa* is about 10-fold of that of *C. aromatica*. On the other hand, *C. aromatica* has much more essential oil components than *C. longa* [6]. Some essential oil components, such as *β*-bisabolene, *β*-sesquiphellandrene and ar-curcumene have been reported and it was showed that these essential oils exhibited multiple biological activities [7, 8]. Recent research further indicated the chemoprotective effects of *C. aromatica* oil on esophageal carcinogenesis [9]. Curdione (Figure 1) of germacrone type is a main ingredient of the essential oils [10]. Recently, Oh et al. [11] indicated that curdione may be a candidate for anti-inflammatory and cancer chemopreventive agents.

As herbal medicinal products have become prevalent throughout the world, the fatalness of herb-drug interactions stands out [12, 13]. Cytochrome P450 (CYP) 3A4 enzyme is the most abundant in human cytochromes. It accounts for up to 30–40% of gastrointestinal CYPs and 50% of intestinal CYPs content. Moreover, CYP3A4 is responsible for the metabolism of *∼*60% of the drugs in current clinical use [14]. Many herbal drugs have been reported as CYP3A4 inducer/inhibitor [15]. Some clinical trials also showed that various herbal products can affect the metabolism of drugs which are CYP3A4 substrate [16, 17]. The inhibition/induction of CYP3A4 has been reported as a significant reason for herb-drug interaction [18].

The Caco-2 cell model is a well-established model to study the absorption and related mechanism of drugs [19–21]. Schmiedlin-Ren et al. [22] showed that expression of CYP3A4 in Caco-2 cells was up-regulated by 1-alpha, 25 dihydroxyvitamin D_3_ (1*α*,25-(OH)_2_-D_3_) treatment, and that these cells might be a promising model to simulate the absorption and metabolism of small intestine. Following that, some researches had verified the activity level of CYP3A4 in the modified Caco-2 cells and showed that the expression and activity of CYP3A4 were inhibited by grapefruit juice [23–25]. This modified cell has become an accepted tool to detect inhibition/induction of CYP3A4. In another of our study, we reported the CYP3A4 inhibitory activity of *C. longa* and curcumin [26]. The present study was conducted to evaluate the effect of *C. aromatica* on CYP3A4, and to identify CYP3A4-inhibiting components in *C. aromatica*. In addition, efforts were tried to clarify the CYP3A4-inhibiting mechanism.

## 2. Methods

### 2.1. Materials and Chemicals

Caco-2 cells (HTB37) were obtained at passage 18 from American Type Culture Collection (Manassas, VA). Caco-2 cell clone P27.7 was a genourous gift from Dr. Paul B. Watkins. *Curcuma aromatica* rhizomes produced from Okinawa pref. were purchased from Nakazen Co., Ltd. (Okinawa, Japan) and correctly identified by the molecular biological method previously reported [27]. This drug sample is preserved with specimen reference number 25343 in the Museum of Materia Medica, Institute of Natural Medicine, University of Toyama, Japan. Curcumin was isolated from the methanol extract of *C. longa* rhizomes by column chromatography and preparative TLC, and curdione was isolated from the methanol extract of *C. aromatica* rhizomes by preparative HPLC. The isolated compounds were identified by comparison of NMR and mass spectral data with those reported [28]. Cychloheximide (CHX), non-essential amino acids (NEAA), antibiotic-antimycotic mixed stock solution, glucose, dl-*α*-tocopherol, sodium selenite, zinc sulfate, ferrous sulfate, ethlenediaminertetracetic acid (EDTA) and trypsin were obtained from Nacalai Tesque (Kyoto, Japan). Fetal bovine serum (FBS) was from Biowest, Inc. (Nuaiillé, France). Testosterone, 6*β*-OH testosterone, nifedipine and oxidized nifedipine were purchased from Sigma Chemical Co. (St. Louis, MO, USA). Dulbelcco's modified Eagle's medium (DMEM), 1*α*, 25-(OH)_2_-D_3_ were from Wako Pure Chemical Industries. Transwell polycarbonate cell culture inserts (24 mm diameter, 0.4 *μ*M pore size) were from Costar Corp. (Bedford, MA, USA). Millicell ERS device was obtained from Millipore (Bedford, MA, USA). Stock solution of the methanol extract of *C. aromatica* and its hexane soluble fraction, curcumin and curdione were prepared at 50 mg ml^−1^ and 100 mM in DMSO and stored in −20°C.

### 2.2. Cell Culture Conditions

Caco-2 cells at passages 24–27 and Caco-2 clone cells at passages 24–30 were used for all experiments. Cell cultures were maintained in a humidified 37°C incubator with 5% carbon dioxide in air atmosphere. Caco-2 cells were grown in plastic tissue culture dishes in a maintenance medium consisting of DMEM containing 25 mM glucose, 4 mM l-glutamine, 0.1 mM nonessential amino acids, 100 units ml^−1^ penicillin, 100 units ml^−1^ streptomycin, 250 nM amphotericin and supplemented with 10% heat-inactivated FBS. When the cells reached 80% confluence, they were removed using 0.2% trypsin/EDTA, diluted with 1 : 4 and reseeded onto fresh tissue culture dishes. The medium was changed at an interval of 2-3 days.

### 2.3. Preparation of Methanol Extract and Hexane Fraction

*Curcuma aromatica* rhizome was pulverized and 10 g of the powder was extracted with 150 ml of methanol under reflux conditions for 3 hours. The organic solvent was evaporated *in vacuo* to give an extract. Of the extract, 567 mg was dissolved in 30 ml of methanol and extracted three times with 30 ml of hexane to separate the methanol extract into hexane and methanol soluble fractions (164 and 282 mg, resp.). The curcumin content of the methanol extract was analyzed as previously described [26].

### 2.4. GC-MS Analysis

Curdione contents of the methanol extract and hexane fraction were analyzed using a Shimadzu GC-17A gas chromatograph interfaced with a Shimadzu QP-5000 quadrupole mass spectrometer (Shimadzu Co., Kyoto, Japan). The GC/MS system was operated with an interface temperature of 280°C and an ion source temperature of 280°C. EI mass spectra were obtained at an ionizing energy of 70 eV and eission current of 60 *μ*A. Chromatographic separation was achieved using a DB-1MS fused-silica capillary column (30 m × 0.25 mm i.d., film thickness 0.1 *μ*m; J&W Scientific, Folsom, CA, USA). Helium was used as a carrier gas at a flow rate of 1 ml min^−1^. An AOC-20i autoinjector (Shimadzu Co.) was used to inject 2 *μ*l of standard solution, extract or fraction into the GC/MS system. The gas chromatograph was equipped with a split/splitless injection port operated at 260°C. The samples were injected in the split mode (split ratio 1 : 20). The GC oven temperature was initially held at 100°C for 2 min and then temperature-programmed at 10°C min^−1^ to 280°C.

### 2.5. Three-Dimensional HPLC Analysis

The methanol extract and hexane fraction were dissolved in methanol and then submitted for HPLC analysis with a Shimadzu LC-20A system equipped with a Shimadzu SPD M10A photodiode array detector. A Waters Symmetry C18 column (150 mm × 4.6 mm i.d., 5 *μ*m) was used. The column temperature was set at 40°C and eluted compounds were detected by monitoring at 200–500 nm. The mobile phase was a binary eluent of (A) 5 mM ammonium acetate solution (pH 3.6), (B) CH_3_CN under following gradient conditions: 0–30 min linear gradient from 10 to 100% B, 30–40 min isocratic at 100% B. The flow rate was 1 ml min^−1^.

### 2.6. CellTiter 96 Aqueous Cell Proliferation Assay (Promega)

The cytotoxicity of methanol extract, hexane fraction and curdione on Caco-2 clone cell proliferation was determined using CellTiter 96 aqueous one solution reagent (Promega Corporation). Caco-2 cells at a concentration of 10^5^ cells cm^−2^ were seeded in 96-well plates. After the cells had reached 80–90% confluence, they were treated with 0–0.5 mg ml^−1^ extract/fraction or 0–500 *μ*M curdione for 72 hours. Following removal of the drugs, cells were washed with phosphate-buffered saline (PBS). The cells were then incubated in the serum-free maintenance medium (100 *μ*l) with one solution reagent (20 *μ*l) for a further 4 hours. Viability was defined as the ratio (expressed as a percentage) of absorbance of treated cells to untreated cells at 490 nm.

### 2.7. 1*α*,25-(OH)_2_-D_3_ Treatment

Caco-2 cells were seeded at 5×10^5^ cells cm^−2^ onto the culture insert and cultured in complete growth medium, which was prepared based on the maintenance medium supplied with 45 nM dl-*α*-tocopherol and 20% heat-inactived FBS. After 10 days, the medium was additionally supplemented with sodium selenite (0.1 *μ*M), zinc sulfate (3 nM), ferrous sulfate (5 *μ*M) and 1*α*,25-(OH)_2_-D_3_ (250 nM). Simultaneously, the content of FBS was decreased to 5%. Cells were incubated for further 3 weeks.

### 2.8. Monolayer Integrity

Cell monolayer integrity was evaluated with transepithelial electrical resistance (TEER). Resistance was measured using a Millicell-ERS resistance system after the medium was changed. An insert that did not contain cells was used as background resistance. TEER was calculated from the background-corrected resistance and the surface area of the insert (4.76 cm^2^).

### 2.9. Inhibition of CYP3A4 Catalytic Activity

After treated with 1*α*,25-(OH)_2_-D_3_ for 3 weeks, extract/fraction, curcumin or curdione was added to the apical compartment and the incubation lasted for 72 hours. After the medium was aspirated, the monolayers were washed with the warm EBSS buffer twice. The EBSS buffer containing nifedipine (200 *μ*M) was added to the apical compartment. After incubating the cells at 37°C for 4 hours, basolateral (2.6 ml) media was collected and stored at −80°C for analysis.

### 2.10. Analysis of Oxidized Nifedipine by HPLC

Ethylacetate (EtOAc, 3 ml) was added to media (1 ml) in an amber vial. Then, the content of each vial was mixed using a vortex device and the two layers were separated by centrifugation at 1500 g for 10 min. From each upper organic layer, 2.5 ml was transferred to another amber vial and dried using a centrifugation evaporator. The content was dissolved in 200 *μ*l of eluate (MeOH:H_2_O = 55 : 45) and 50 *μ*l was injected to the HPLC system. The reversed-phase separation was performed in Inertsil ODS-3 column (150 mm × 4.6 mm i.d., 5 *μ*m particles, GL Sciences Inc., Japan). Elution was performed at a rate of 1 ml min^−1^ and monitored at 254 nm. Standards of oxidized nifedipine were made in methanol. Concentrations were obtained by extrapolation of the peak area from a standard curve.

### 2.11. Cell Lysis

Cell monolayers were washed with cold PBS for three times. Total cellular proteins were extracted in the lysis buffer containing 150 mM NaCl, 10 mM Tris (pH 7.4), 1 mM EDTA, 1% Triton X-100, 1% deoxycholic acid and protease inhibitor mixture. Protein concentrations were determined by the BCA protein assay reagent kit (Sigma-Aldrich), using bovine serum albumin as a standard. Total cell lysate was stored at −80°C for analysis.

### 2.12. Western Blot Analysis

Proteins (12 *μ*g) from the total cell lysate were analyzed by SDS–PAGE (12.5% gel). After blotting, the PVDF transfer membrane (Amersham Biosciences) was blocked with 5% skimmed milk in PBS with Tween 20 at room temperature for 1 hour. Immunoblots were incubated at room temperature for 1 hour with the specific primary antibody to CYP3A4 (BD Biosciences). After further washing, the membranes were incubated for 1 hour with the secondary antibody (Santa Cruz Biotechnology). Blots were reprobed with antibody to GAPDH as a loading control. Quantitative analysis of immunoblotted bands was performed by a computer program (NIH Image, version 1.61).

### 2.13. RNA Extraction and SYBR GREEN I Real-Time PCR

The total RNA was extracted from the treated cells using TRIzol reagent (QIAGEN). First strand cDNA was generated from 1 *μ*g of total RNA by the oligo(dT) first strand primer (Invitrogen). Real-time PCR was performed with 50 000 fold diluted SYBR GREEN I dye (Invitrogen). For CYP3A4, the forward primer sequence used was 5′-ACTGAGTCCCACAAAGCTCTGTC-3′ and the reverse primer sequence used was 5′-AACTGCATCAATTTCCTCCTGC-3′; for *β*-actin, the forward primer sequence used was 5′-GGTCATCACCATTGGCAATGA-3′ and the reverse primer sequence used was 5′-GTAGTTTCGTGGATGCCACAGG-3′. Aliquots of the reverse-transcription reaction mixture (1 *μ*l) were amplified and detected using a ABI PRISM 7000 Sequence Detector System (Applied Biosystems) with the following profile: 1 cycle of 50°C for 2 min, 1 cycle of 95°C for 5 min and 45 cycles each of 95°C for 15 s and 60°C for 1 min. The CYP3A4 mRNA levels were normalized relative to the *β*-actin mRNA level in each sample.

### 2.14. Statistics

Experiments were repeated for at least three times, and data were expressed as means ± standard deviation (SD). Statistical significances between treatment groups were determined using Student's *t*-test or ANOVA and Scheffe's test. Treatment was considered significantly different from controls if *P* < .05.

## 3. Results

### 3.1. Induction of the CYP3A4 Activity of Caco-2 Clone Cells by 1*α*,25-(OH)_2_-D_3_

**Figure 2:**
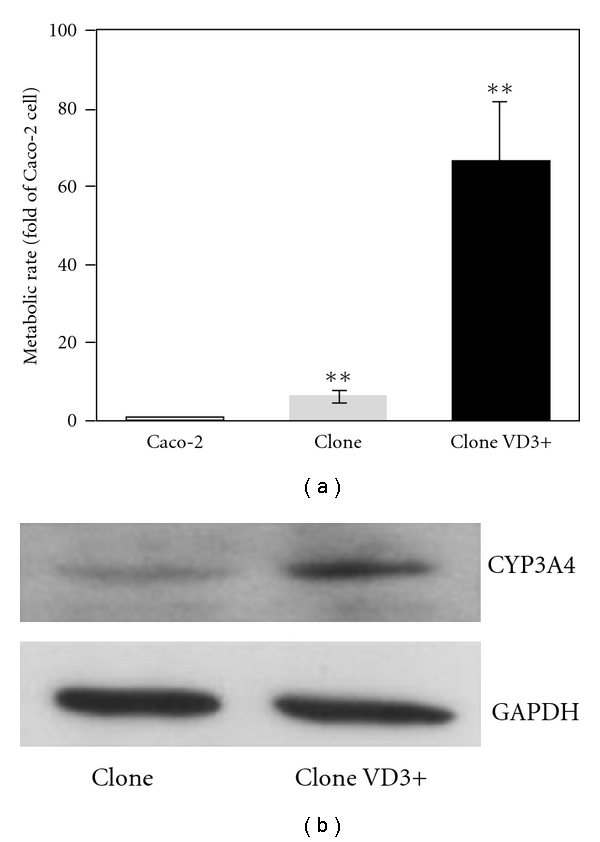
Induction of CYP3A4 activity and protein expression by 1*α*,25-(OH)_2_-D_3_. Caco-2 clone cells grown on culture inserts were treated with 1*α*,25-(OH)_2_-D_3_ (250 nM) for 3 weeks after confluence. (a) Metabolic activity of 1*α*,25-(OH)_2_-D_3_-treated Caco-2 clone cells. Nifedipine (200 *μ*M) was added to the apical compartment. After 4 hours, basolateral media was collected and analyzed for oxidized nifedipine. Results are means ± SD from triplicate experiments. **P < .01 versus control. (b) CYP3A4 expression level of 1*α*,25-(OH)_2_-D_3_-induced Caco-2 clone cells.

Caco-2 cells from ATCC were incubated for 4 weeks without 1*α*,25-(OH)_2_-D_3_ treatment. Then nifedipine was ultilized to detect the metabolic activity of CYP3A4 [29]. Our pre-experiment indicated that the formation of oxidized nifedipine by CYP3A4 in Caco-2 cells was correlated with the concentration of nifedipine added in the apical compartment when the concentration of nifedipine was below 200 *μ*M. Thus, in our experiment, nifedipine with a concentration of 200 *μ*M was used. Nifedipine (200 *μ*M) was added to the apical compartment and little oxidized nifedipine was detected in the basolateral side after incubation for 4 hours. As shown in Figure 2, Caco-2 clone cells showed about 6-fold higher metabolic activity of nifedipine than Caco-2 cells. Furthermore, treatment with 1*α*,25-(OH)_2_-D_3_ for 3 weeks increased the nifedipine metabolic activity of Caco-2 clone cells for about 10-fold and oxidized nifedipine was detected in the basolateral compartment as a concentration of 4.6 ± 1.3 *μ*M. The metabolic activity of 1*α*,25-(OH)_2_-D_3_-treated Caco-2 clone cells was induced by *∼*60-fold compared to that of ATCC Caco-2 cells. Moreover, the induction of CYP3A4 protein expression in Caco-2 clone cells was also proved by western blot. The metabolic activity of nifedipine kept stable among different passages of Caco-2 clone cells used in the experiments. Experiments were conducted on monolayers with transepithelial electrical resistance (TEER) of >400 Ω cm^2^.

### 3.2. Methanol Extract, Hexane Fraction, Curcumin and Curdione Inhibited Nifedipine Oxidization in 1*α*,25-(OH)_2_-D_3_-Treated Caco-2 Clone Cells

After treatment for 72 hours, both methanol extract and hexane fraction inhibited cell growth in a concentration-dependent way. They showed no cytotoxicity to Caco-2 cells when the concentration was <0.1 mg ml^−1^. The 50% cytotoxicity concentrations (CC50) of methanol extract and hexane fraction were 0.26 and 0.22 mg ml^−1^, respectively. The CC50 of curcumin was 73 *μ*M (0.027 mg ml^−1^). On the other hand, curdione showed no cytotoxicity to Caco-2 clone cells even when the concentration was as high as 500 *μ*M (0.1182 mg ml^−1^).

**Figure 3:**
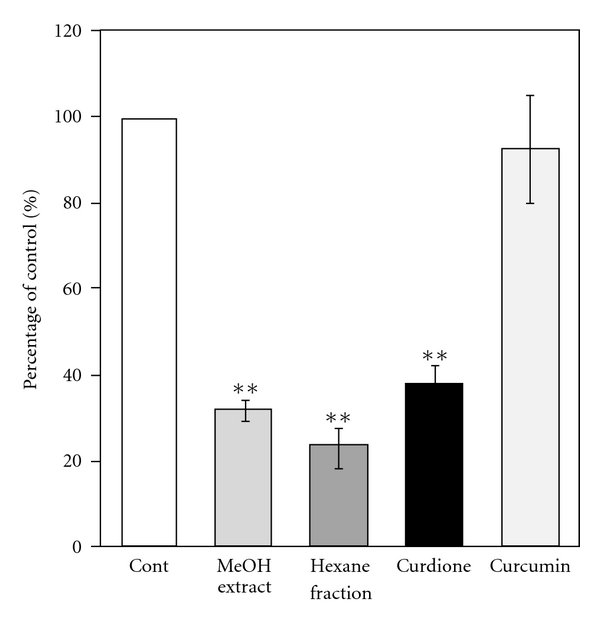
Inhibition of nifedipine oxidation by components from *C. aromatica* (MeOH extract: 0.1 mg ml^−1^; haxane fraction: 0.1 mg ml^−1^; curdione: 20 *μ*M; curcumin: 4 *μ*M). Components were applied to the apical compartment and incubated for 72 hours. After removing components, nifedipine (200 *μ*M) was added to the apical compartment and incubated for 4 hours. The amount of oxidized nifedipine in the basolateral compartment was measured. Results are means ± SD from triplicate experiments. **P < .01 versus control.

**Figure 4:**
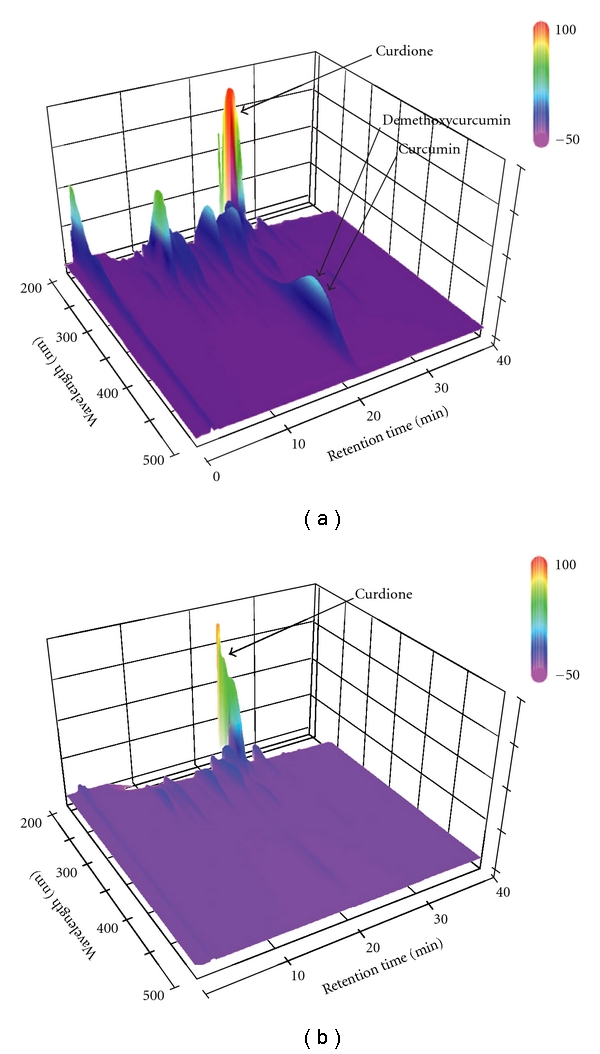
3D-HPLC chromatogram of MeOH extract (a) and hexane fraction (b) of *C. aromatica*.

After Caco-2 clone cells were treated with all drugs for 72 hours, there were no significant changes in TEER. In this study, Caco-2 cells were treated with drugs with various concentrations to calculate the IC50. Then some concentrations without cytotoxicity were picked out for further experiment as follows: methanol extract: 0.1 mg ml^−1^; hexane fraction: 0.1 mg ml^−1^; curdione: 20 *μ*M (0.0047 mg ml^−1^); curcumin: 4 *μ*M (0.0015 mg ml^−1^). In 0.1 mg ml^−1^ of methanol extract, the curcumin concentration is 4 *μ*M since it occupied 1.2% of the methanol extract in content, and the curdione concentration is *∼*40 *μ*M because it occupied 11.4% of the methanol extract. Because of the strong inhibitory activity of curdione on oxidized nifedipine formation, half of the curdione concentration in methanol extract was picked out as 20 *μ*M. The results were indicated in Figure 3. The amount of oxidized nifedipine formed by methanol extract-treated Caco-2 monolayer was *∼*32% of that of the control monolayer, and just *∼*23% of oxidized nifedipine was detected in the hexane fraction treated well compared to that in the control well. Curdione (20 *μ*M) also inhibited *∼*60% of oxidized nifedipine formation. Curcumin (4 *μ*M) showed no significant inhibitory effects on the metabolism of nifedipine. 3D-HPLC chromatograms of methanol extract and hexane fraction were shown in Figure 4. The result indicated that almost all curdione content in methanol extract was fractionated by hexane. The IC50 of hexane fraction to nifedipine oxidization was 14 *μ*g ml^−1^, which is *∼*67% of the value for methanol extract. The content in methanol extract and IC50 of curdione were 11.4% and 3.9 *μ*g ml^−1^ (16.86 *μ*M), respectively. On the other hand, IC50 of curcumin was >11.1 *μ*g ml^−1^ (30 *μ*M) (Table 1).

### 3.3. Methanol Extract, Hexane Fraction, Curcumin and Curdione Decreased the Expression Level of CYP3A4 Protein but Had No Effect on the Expression of CYP3A4 mRNA in 1*α*,25-(OH)_2_-D_3_-Treated Caco-2 Clone Cells

**Figure 5:**
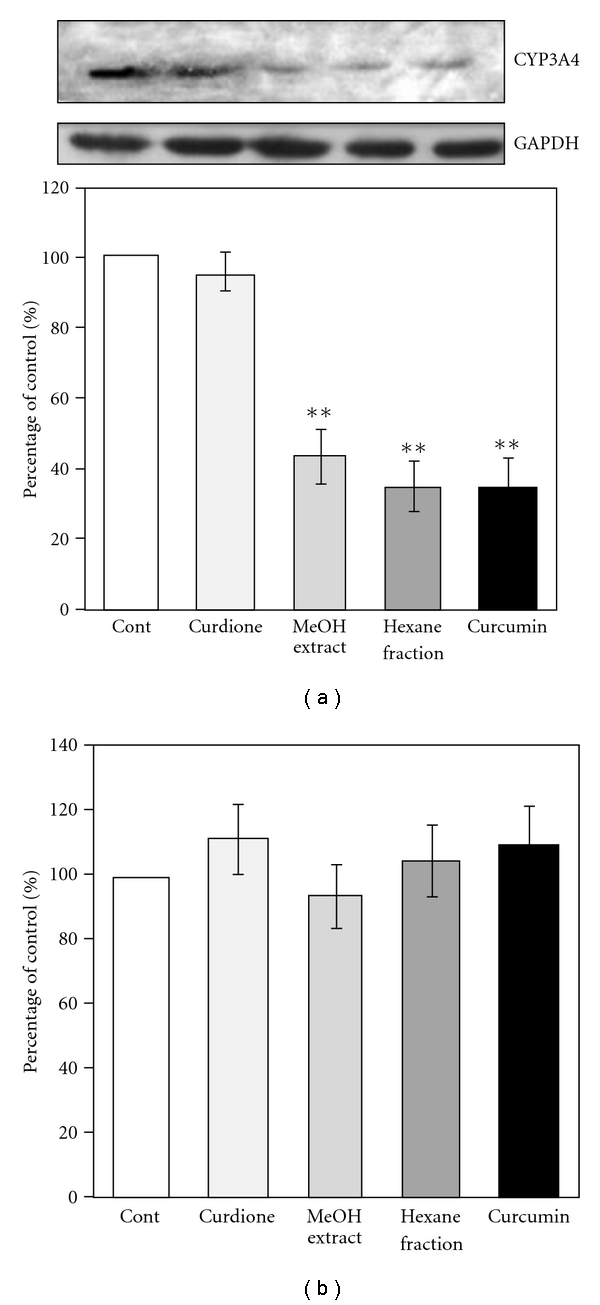
Effects of MeOH extract, hexane fraction, curcumin and curdione on CYP3A4 protein and mRNA expression levels. Components were applied to the apical compartment and incubated for 72 hours. Then cell lysate or total RNA was prepared for western blot or real-time PCR. (a) Representative western immunoblot for CYP3A4 and quantitative analysis of CYP3A4 immunoprotein. The western immunoblot band intensities were normalized with that of GAPDH. Results are means ± SD from triplicate experiments. **P < .01 versus control. (b) Quantitative analysis of CYP3A4 mRNA. Results are means ± SD from triplicate experiments.

To clarify the CYP3A4 inhibitory mechanism of methanol extract, hexane fraction, curcumin and curdione, western blot and real-time PCR were carried out (Figure 5). When Caco-2 clone cells were treated with methanol extract, hexane fraction and curdione for 72 hours, the CYP3A4 protein expression was decreased significantly to 30–50% compared with that of the control group. Curcumin treatment showed no affect on CYP3A4 protein expression. On the other hand, no change was found in the CYP3A4 mRNA expression levels when Caco-2 clone cells were treated with any of them.

**Figure 6:**
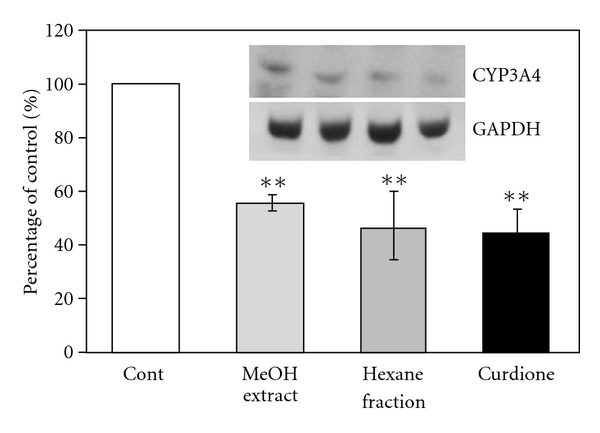
Effects of MeOH extract, hexane fraction and curdione on CYP3A4 protein expression in CHX-treated Caco-2 cells. The western immunoblot band intensities were normalized with that of GAPDH. Results are means ± SD from triplicate experiments. **P < .01 versus control.

After 1**α**,25-(OH)_2_-D_3_ treatment, CHX (10 *μ*g ml^−1^) was added to Caco-2 clone cells. After 1 hour, methanol extract (0.1 mg ml^−1^), hexane fraction (0.1 mg ml^−1^) and curdione (20 *μ*M) were added and the treatment lasted for 24 hours. Western blot was used to evaluate the variation of the CYP3A4 protein expression level. Compared to the control group which is treated just with CHX, all of methanol extract, hexane fraction and curdione treatment decreased the protein expression of CYP3A4 by 30–60% (Figure 6).

## 4. Discussion

*Curcuma* species (*C. longa*, *C. zedoaria* and *C. aromatica)* are used as traditional medicines and supplements in East Asia. In the past, *Curcuma*s were recommended as botanical supplements and traditional herbs with a high rank. It was thought that *Curcumas* would not lead to any side-effect even when they were administrated with a very large dose. However, since 1996, case reports appeared and showed that *Curcumas* administration had caused some side-effect, such as drug-induced hepatitis [30]. It became necessary to offer some information for the correct use of *Curcumas.* Nagamura et al. [31] reported that *C. longa* and curcumin inhibited the function of both sulfotransferase (SULT) and UDP-glucuronosyl transferase (UGT) activity in Caco-2 cells. In our previous report, we have described that *C. longa* and *C. zedoaria* might inhibit the catalytic activity of intestinal CYP3A4, and curcumin was not the major compound responsible for this inhibitory effect [28]. In *Curcuma* species, *C. aromatica* (Haruukon in Japanese) is a special member since there is confusion in the origin of this crude drug and the nomenclature of this plant is inconsistent in East Asia. In this study, *C. aromatica* (Haruukon in Japanese) identified by the molecular biological method were used and we reported for the first time that *C. aromatica* might inhibit the intestinal CYP3A4 activity. Furthermore, we proved that curdione, an essential oil from *C. aromatica*, played an important role in the inhibitory activity. We even identified that curdione might inhibit CYP3A4 activity by accelerating the degradation of CYP3A4 protein. To our knowledge, this is the first of these reports.

Almost all herbs are administrated orally. The first major barrier for them to cross is the intestinal epithelium. In our previous study, 1*α*,25-(OH)_2_-D_3_-modified Caco-2 cells had been used to mimic the absorption and metabolism of small intestine since this system showed ample CYP3A4 metabolic activity. However, we found that sometimes the CYP3A4 activity level was labile among different Caco-2 monolayers although we had tried to keep the incubation condition identical. To obtain genetic homogeneity among the cells, Caco-2 clone cells (P27.7) were employed in these experiments instead of normal Caco-2 cells. Schmiedlin-Ren et al. [22] reported that the midazolam 1′-hydroxylation rate was 75.9 pmol min^−1^ g^−1^ of cells when the clone cells were treated with 250 nM 1*α*,25-(OH)_2_-D_3_ for 2 weeks. Some researches reported that the CYP3A4 activity can be improved by *∼*100-fold in CYP3A4-transfected Caco-2 cells [32, 33]. However, there is limitation in these cells when they are utilized to clarify the mechanism of herb-drug interaction. In our experiment, we proved that the Caco-2 clone cells had a relatively high level of CYP3A4 activity compared to our previously used Caco-2 cells, and that 1*α*,25-(OH)_2_-D_3_ treatment would significantly increase the CYP3A4 activity when nifedipine was used as a substrate. Most importantly, the CYP3A4 activity of our clone cells kept stable during our experiments.

Using 1*α*,25-(OH)_2_-D_3_-treated Caco-2 clone cells, we found that the metabolic activity and protein expression of CYP3A4 were markedly inhibited when the cells were treated with 0.1 mg ml^−1^ methanol extract of *C. aromatica* for 72 hours. Our result showed that 4 *μ*M curcumin had no inhibitory affect on CYP3A4 metabolic activity. Curcumin had no relation with the CYP3A4 inhibitory activity of *C. aromatica*. Similarly, although some papers reported that curcumin might inhibit the metabolic activity of CYP3A, the effect was very weak [34–36]. Price et al. reported that the IC50 of curcumin was 29 *μ*M when curcumin was directly added to human liver microsome. Besides curcumin, some essential oils have been isolated from *C. aromatica*. A hexane fraction, which occupied 49% of the methanol extract content, showed *∼*2-fold stronger inhibitory activity to CYP3A4 activity compared to methanol extract. Furthermore, we found that curdione, which occupied 11.4% of the methanol extract, significantly inhibited the activity of CYP3A4 with IC50 as 3.9 *μ*g ml^−1^. Theoretically, if curdione was the sole component that was responsible for the CYP3A4 inhibitory activity, its IC50 should be *∼*2.4 *μ*g ml^−1^, since the IC50 of methanol extract was 21 *μ*g ml^−1^. The relationship between CYP3A4 inhibitory activity and content in the MeOH extract of each drugs is summarized in Table 1. Possibly, it is curdione that inhibits the CYP3A4 activity in methanol extract. Furanocomarins as mediators of the interaction of grapefruit juice and the model CYP3A4 substrate using furanocoumarin-free grapefruit juice have been established [37]. Further study might become necessary to verify the key role of curdione in the CYP3A4 inhibitory effect of *C. aromatica* using a similar idea.

The decreased Caco-2 CYP3A4 protein levels observed after *C. aromatica* treatment implied that the interaction was not simple competitive inhibition. Same as *C. longa* and *C. zedoaria*, *C. aromatica* treatment did not decrease the expression level of CYP3A4 mRNA. Curdione showed the same CYP3A4 inhibitory effect as *C. aromatica*. The results indicated that *C. aromatica*/curdione did not decrease CYP3A4 protein content by a transcriptional way. Rather, cychroheximide, a protein synthesis inhibitor was used to clarify the further mechanism. In our experiment, a concentration as 10 *μ*g ml^−1^ was used since it has been reported that 98% of protein synthesis would be inhibited on this concentration [38]. We found that *C. aromatica*/curdione decreased the expression level of CYP3A4 protein at least partially by accelerating the degradation of CYP3A4 protein. Our results indicated that the inhibitory pattern of *C. aromatica* was very similar to that of grapefruit juice, which exhibited a mechanism-based inhibitory effect on intestinal CYP3A4. The return of CYP3A4 activity would require *de novo* enzyme synthesis, which would lead to prolonged inhibitory effect.

Sharma et al. [39] reported that *Curcuma* extract (curcuminoid: essential oil = 10:1) could be administrated safely at doses of up to 2.2 g per day. Taking the gastric fluid volume to be 1–3 l for an adult human with the body weight of 60 kg [40], the concentrations of *Curcuma* drugs in the gastric fluid would be 0.7–2.2 mg ml^−1^, respectively. In Japan, the powder of *Curcuma aromatica* is recommended as a supplement and the recommended dose ranges from 2 g per day to 5 g per day. Normally, *C. aromatica* has a curdione content as *∼*0.65%. Thus, the curdione in the gastric fluid would be 18.3–137 4 *μ*M. It is possible for administrated *Curcuma* drugs and curdione to affect the pharmacokinetics of co-administrated drugs by interfering with the intestinal CYP3A4 function.

In summary, we provide strong evidence that *C. aromatica* might inhibit the activity of intestinal CYP3A4. We further showed that curdione, a major constituent of *C. aromatica*, might be responsible for this inhibitory effect. We also indicated that curdione might inhibit the activity of CYP3A4 by increasing its degradation. Further studies in humans are needed to clarify the impact of this nutrient on metabolism of orally administrated substrates of CYP3A4, and possible other drug-metabolizing enzymes.

## Funding

Mukogawa Women's University.

## Figures and Tables

**Table 1:** Relationship between CYP3A4 inhibitory activity and content in MeOH extract.

	IC_50_ (*μ*g/ml)	Content (%)
MeOH extract	21 ± 1.8	—
Hexane fraction	14 ± 2.6 (11.3^(a)^)	54.0
Curdion	3.9 ± 1.7 (2.4^(a)^)	11.4
Curcumin	>11.1	1.2

^(a)^Theoritical value calculated from the content in MeOH extract hypothesizing that curdion is the only component responsible for the CYP3A4 inhibitory activity of *C. aromatica* when the IC_50_ of MeOH extract is 21 *μ*g/ml. Results are means ± SD from triplicate experiments.

## References

[1] Araújo CAC, Leon LL (2001). Biological activities of *Curcuma longa* L. *Memorias do Instituto Oswaldo Cruz*.

[2] Matsuda H, Ninomiya K, Morikawa T, Yoshikawa M (1998). Hepatoprotective, superoxide scavenging, and antioxidative activities of aromati constituents from the bark of betula platyphylla var japonica. *Bioorganic & Medicinal Chemistry Letters*.

[3] Maheshwari RK, Singh AK, Gaddipati J, Srimal RC (2006). Multiple biological activities of curcumin: a short review. *Life Sciences*.

[4] Thangapazham RL, Sharma A, Maheshwari RK (2006). Multiple molecular targets in cancer chemoprevention by curcumin. *AAPS Journal*.

[5] Salvioli S, Sikora E, Cooper EL, Franceschi C (2007). Curcumin in cell death processes: a challenge for CAM of age-related pathologies. *Evidence-Based Complementary and Alternative Medicine*.

[6] Minami M, Nishio K, Ajioka Y (2009). Identification of Curcuma plants and curcumin content level by DNA polymorphisms in the trnS-trnfM intergenic spacer in chloroplast DNA. *Journal of Natural Medicines*.

[7] Choochote W, Chaiyasit D, Kanjanapothi D (2005). Chemical composition and anti-mosquito potential of rhizome extract and volatile oil derived from *Curcuma aromatica* against Aedes aegypti (Diptera: Culicidae). *Journal of Vector Ecology*.

[8] Lai EYC, Chyau C-C, Mau J-L (2004). Antimicrobial activity and cytotoxicity of the essential oil of *Curcuma zedoaria*. *American Journal of Chinese Medicine*.

[9] Li Y, Wo JM, Liu Q, Li X, Martin RCG (2009). Chemoprotective effects of *Curcuma aromatica* on esophageal carcinogenesis. *Annals of Surgical Oncology*.

[10] Kojima H, Yanai T, Toyota A (1998). Essential oil constituents from Japanese and Indian *Curcuma aromatica* rhizomes. *Planta Medica*.

[11] Oh O-J, Min H-Y, Lee SK (2007). Inhibition of inducible prostaglandin E2 production and cyclooxy-genase-2 expression by curdione from *Curcuma zedoaria*. *Archives of Pharmacal Research*.

[12] Mills E, Montori V, Perri D, Phillips E, Koren G (2005). Natural health product-HIV drug interactions: a systematic review. *International Journal of STD and AIDS*.

[13] Fugh-Berman A, Ernst E (2001). Herb-drug interactions: review and assessment of report reliability. *British Journal of Clinical Pharmacology*.

[14] Guengerich FP (1999). Cytochrome P-450 3A4: regulation and role in drug metabolism. *Annual Review of Pharmacology and Toxicology*.

[15] Qiu F, Zhang R, Sun J (2008). Inhibitory effects of seven components of danshen extract on catalytic activity of cytochrome P450 enzyme in human liver microsomes. *Drug Metabolism and Disposition*.

[16] Andrén L, Andreasson A, Eggertsen R (2007). Interaction between a commercially available St. John's wort product (Movina) and atorvastatin in patients with hypercholesterolemia. *European Journal of Clinical Pharmacology*.

[17] Gurley BJ, Gardner SF, Hubbard MA (2005). Clinical assessment of effects of botanical supplementation on cytochrome P450 phenotypes in the elderly: St John’s Wort, garlic oil, Panax ginseng and Ginkgo biloba. *Drugs and Aging*.

[18] Wrighton SA, Schuetz EG, Thummel KE, Shen DD, Korzekwa KR, Watkins PB (2000). The human CYP3A subfamily: practical considerations. *Drug Metabolism Reviews*.

[19] Hilgers AR, Conradi RA, Burton PS (1990). Caco-2 cell monolayers as a model for drug transport across the intestinal mucosa. *Pharmaceutical Research*.

[20] Neuhaus W, Bogner E, Wirth M (2006). A novel tool to characterize paracellular transport: the APTS-dextran ladder. *Pharmaceutical Research*.

[21] Hou X-L, Takahashi K, Tanaka K (2008). Curcuma drugs and curcumin regulate the expression and function of P-gp in Caco-2 cells in completely opposite ways. *International Journal of Pharmaceutics*.

[22] Schmiedlin-Ren P, Thummel KE, Fisher JM, Paine MF, Lown KS, Watkins PB (1997). Expression of enzymatically active CYP3A4 by Caco-2 cells grown on extracellular matrix-coated permeable supports in the presence of 1*α*,25- dihydroxyvitamin D3. *Molecular Pharmacology*.

[23] Aiba T, Susa M, Fukumori S, Hashimoto Y (2005). The effects of culture conditions on CYP3A4 and MDR1 mRNA induction by 1alpha,25-dihydroxyvitamin D(3) in human intestinal cell lines, Caco-2 and LS180. *Drug Metabolism and Pharmacokinetics*.

[24] Hara H, Yasunami Y, Adachi T (2002). Alteration of cellular phosphorylation state affects vitamin D receptor-mediated CYP3A4 mRNA induction in Caco-2 cells. *Biochemical and Biophysical Research Communications*.

[25] Paine MF, Criss AB, Watkins PB (2005). Two major grapefruit juice components differ in time to onset of intestinal CYP3A4 inhibition. *Journal of Pharmacology and Experimental Therapeutics*.

[26] Hou XL, Takahashi K, Kinoshita N (2007). Possible inhibitory mechanism of curcuma drugs on CYP3A4 in 1 alpha, 25 dihydroxyvitamin D3 treated Caco-2 cells. *International Journal of Pharmaceutics*.

[27] Cao H, Sasaki Y, Fushimi H, Komatsu K (2001). Molecular analysis of medicinally-used Chinese and Japanese *Curcuma* based on 18S rRNA gene and trnK gene sequences. *Biological & Pharmaceutical Bulletin*.

[28] Yan J, Chen G, Tong S, Feng Y, Sheng L, Lou J (2005). Preparative isolation and purification of germacrone and curdione from the essential oil of the rhizomes of Curcuma wenyujin by high-speed counter-current chromatography. *Journal of Chromatography A*.

[29] Hu M, Li Y, Davitt CM (1999). Transport and metabolic characyerization of Caco-2 cells expressing CYP3A4 and CYP3A4 oxidoreductase. *Pharmaceutical Research*.

[30] Kumashiro R, Hisamochi A, Sata M (2006). Liver injury by health food. *Sogo-Rinsho*.

[31] Naganuma M, Saruwatari A, Okamura S, Tamura H (2006). Turmeric and curcumin modulate the conjugation of 1-naphthol in caco-2 cells. *Biological and Pharmaceutical Bulletin*.

[32] Crespi CL, Penman BW, Hu M (1996). Development of Caco-2 cells expressing high levels of cDNA-derived cytochrome P4503A4. *Pharmaceutical Research*.

[33] Hu M, Li Y, Davitt CM (1999). Transport and metabolic characterization of Caco-2 cells expressing CYP3A4 and CYP3A4 plus oxidoreductase. *Pharmaceutical Research*.

[34] Price RJ, Scott MP, Giddings AM (2008). Effect of butylated hydroxytoluene, curcumin, propyl gallate and thiabendazole on cytochrome P450 forms in cultured human hepatocytes. *Xenobiotica*.

[35] Zhang W, Lim L-Y (2008). Effects of spice constituents on P-glycoprotein-mediated transport and CYP3A4-mediated metabolism in vitro. *Drug Metabolism and Disposition*.

[36] Valentine SP, Le Nedelec MJ, Menzies AR, Scandiyn MJ, Goodin MG, Rosengren RJ (2006). Curcumin modulates drug metabolizing enzymes in the female Swiss Webster mouse. *Life Sciences*.

[37] Paine MF, Widmer WW, Hart HL (2006). A furanocoumarin-free grapefruit juice establishes furanocoumarins as the mediators of the grapefruit juice-felodipine interaction. *American Journal of Clinical Nutrition*.

[38] Israel DI, Estolano MG, Galeazzi DR, Whitlock JP (1985). Superinduction of cytochrome P-450 gene transcription by inhibition of protein synthesis in wild type and variant mouse hepatoma cells. *The Journal of Biological Chemistry*.

[39] Sharma RA, McLelland HR, Hill KA (2001). Pharmacodynamic and pharmacokinetic study of oral Curcuma extract in patients with colorectal cancer. *Clinical Cancer Research*.

[40] Han Y, Tan TMC, Lim L-Y (2006). Effects of capsaicin on P-gp function and expression in Caco-2 cells. *Biochemical Pharmacology*.

